# Supplementary material for: From imaging to computational domains for physics-driven molecular biology simulations: Hindered diffusion in platelet masses
Source: PLoS Comput Biol. 2025 Jul 7;21(7):e1012853. doi: 10.1371/journal.pcbi.1012853 (PMC12244541; doi:10.1371/journal.pcbi.1012853)
Supplement: S1 Text — Binning approach for computing localized diffusivities to generate diffusion heatmaps. Fig B in S1 Text. Sample mean-square displacement (MSD) plots for computing localized diffusion coefficients in sparse and dense sub-domains. Green slopes demonstrate the faster rate of diffusion at short time before the effects of the domain are fully felt by particles, while red slopes demonstrate the long-time rate of diffusion. We calculated molecular diffusivity from the MSD curve at t > 0.001 s (black dashed line). (PDF) [file pcbi.1012853.s001.pdf]

S1 Text - SUPPORTING METHODS

Creating Diffusion Heatmaps

To create spatially resolved diffusivity heatmaps, each 10×10 μm^2 domain was subdivided into 81 2×2 μm^2 subdomains staggered by 1 μm in the *x* and *y* dimensions as shown in Fig. A. LKMC simulations were then executed in each of these subdomains to compute the particle diffusivity specific to that region. These diffusivity values were averaged in the regions where subdomains overlapped, providing a total of 100 localized particle diffusivities over the 81 subdomains. All values were normalized by the diffusivity of an unhindered particle of identical size. The same procedure was applied to the 23.6×16.1 μm^2 hybrid domain (Fig. 5 in the main text) yielding a total of 345 subdomains and 384 averaged particle diffusivities.

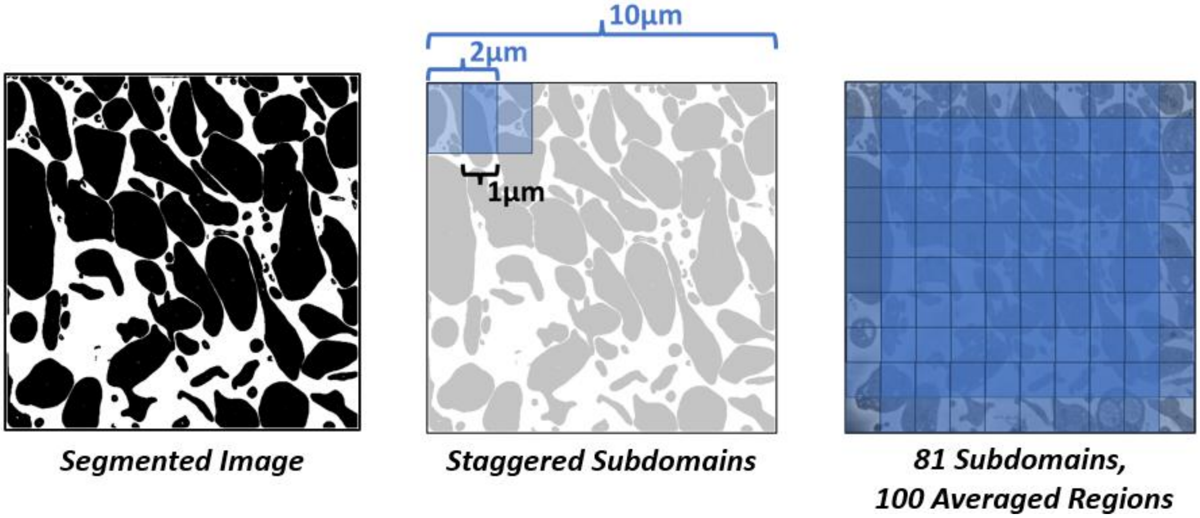

Fig A in S1 Text. Binning approach for computing localized diffusivities to generate diffusion heatmaps.

Mean Squared Displacement vs Time

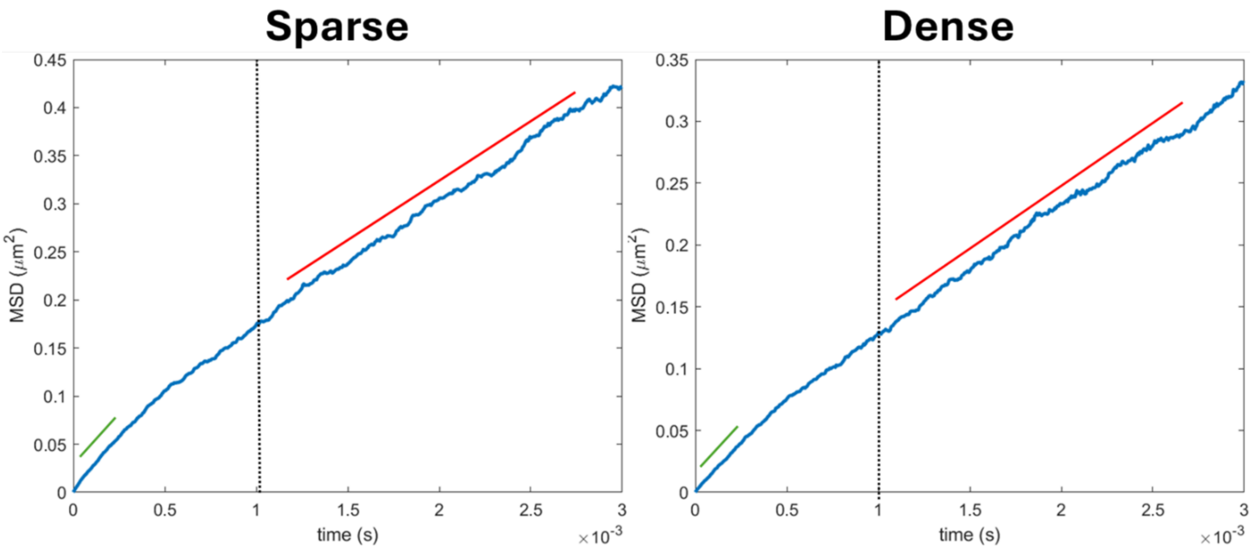

Fig B in S1 Text. Sample mean-square displacement (MSD) plots for computing localized diffusion coefficients in sparse and dense sub-domains. Green slopes demonstrate the faster rate of diffusion at short time before the effects of the domain are fully felt by particles, while red slopes demonstrate the long-time rate of diffusion. We calculated molecular diffusivity from the MSD curve at $t > 0.001$ s (black dashed line).
