# Supplementary material for: From imaging to computational domains for physics-driven molecular biology simulations: Hindered diffusion in platelet masses
Source: PLoS Comput Biol. 2025 Jul 7;21(7):e1012853. doi: 10.1371/journal.pcbi.1012853 (PMC12244541; doi:10.1371/journal.pcbi.1012853)
Supplement: S2 Text — BTV as a function of particle size/LKMC lattice spacing for different domain types. Optimal BTV values (symbols) represent values at which D/DGT=1. Error bars represent the BTV range for 0.9≤D/DGT≤1.1. Green shaded region at each resolution represents the range of BTV that exhibits 0.9≤D/DGT≤1.1 across all domain types, while blue shaded region is the BTV range for which all domain types across all lattice spacings exhibit 0.9≤D/DGT≤1.1. Fig D in S2 Text. The pore width distribution (in nm) for each domain type (sparse, dense, and dense with RBC). (PDF) [file pcbi.1012853.s002.pdf]

S2 Text - SUPPORTING RESULTS

Gradient Flow Optimal BTV and Particle Size

All simulations in this study assume that a particle occupies a single lattice site. Consequently, the lattice spacing, or resolution, in the LKMC simulations effectively sets the size (radius) of a diffusing particle to 8 nm. Here, we consider the impact of particle size on the optimal BTV extracted from gradient flow Cellpose output. Shown in Fig. B are the optimal BTVs for different types of domains at lattice resolutions (i.e., particle sizes) ranging from 8 nm (the default value) to 1 nm. The symbols represent the optimal BTVs, while the error bars represent a 10% deviation of the diffusivity. In other words, the BTV range for each case represents predicted diffusivities in the interval, $0.9 \leq D/D_{GT} \leq 1.1$. The green-shaded interval at each lattice resolution represents the range of BTV that exhibits $0.9 \leq D/D_{GT} \leq 1.1$ across all domain types. The blue-shaded region is the BTV range for which all domain types *across all lattice spacings* exhibit $0.9 \leq D/D_{GT} \leq 1.1$. This range was found to be BTV = 11 - 12. We can therefore conclude that BTV~12 may be applied to threshold gradient flow maps for any type of domain and will provide a good estimate for the diffusivity for any relevant particle size.

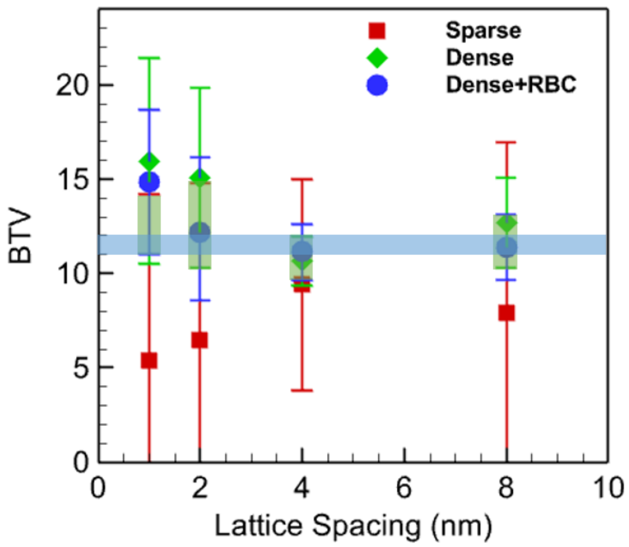

Fig C in S2 Text. BTV as a function of particle size/LKMC lattice spacing for different domain types. Optimal BTV values (symbols) represent values at which $D/D_{GT} = 1$. Error bars represent the BTV range for $0.9 \leq D/D_{GT} \leq 1.1$. Green shaded region at each resolution represents the range of BTV that exhibits $0.9 \leq D/D_{GT} \leq 1.1$ across all domain types, while blue shaded region is the BTV range for which all domain types across all lattice spacings exhibit $0.9 \leq D/D_{GT} \leq 1.1$.

Pore width distribution for each domain type

The distribution of pore widths was evaluated for each domain type (sparse, dense, and dense with RBC). The 'dense' and 'dense with RBC' domain unsurprisingly have very similar pore width distributions, with most pores being 30-50 nm wide. The sparse domain has a much broader distribution of pore widths; most pores have a width <25 nm, but significant fractions have widths in the hundreds of nanometers. All three distributions are right-skewed.

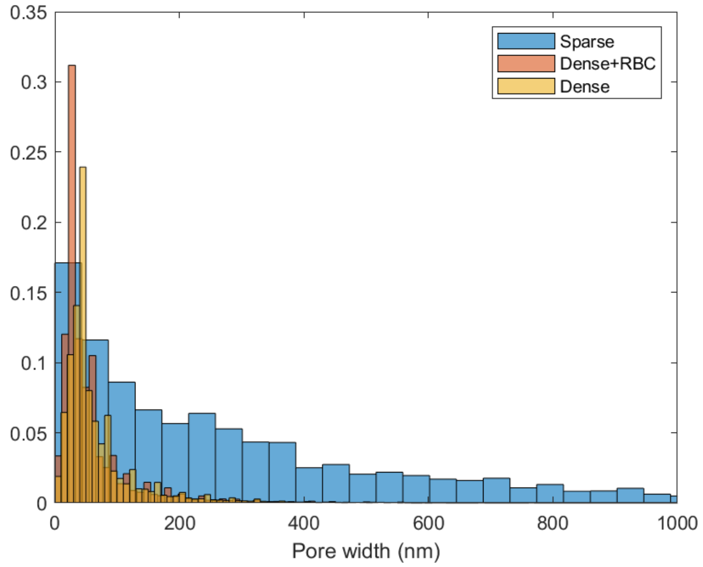

Fig D in S2 Text. The pore width distribution (in nm) for each domain type (sparse, dense, and dense with RBC).
