# Supplementary material for: From imaging to computational domains for physics-driven molecular biology simulations: Hindered diffusion in platelet masses
Source: PLoS Comput Biol. 2025 Jul 7;21(7):e1012853. doi: 10.1371/journal.pcbi.1012853 (PMC12244541; doi:10.1371/journal.pcbi.1012853)
Supplement: S3 Text — The five sparse domains used in our analysis of the optimal BTV of Cellpose’s segmentation outputs. Four versions of each domain are pictured: the original SEM image, the manually segmented ground truth, Cellpose’s gradient flow output, and Cellpose’s cell probability output. Fig F in S3 Text. The five dense domains used in our analysis of the optimal BTV of Cellpose’s segmentation outputs. Four versions of each domain are pictured: the original SEM image, the manually segmented ground truth, Cellpose’s gradient flow output, and Cellpose’s cell probability output. Fig G in S3 Text. The five dense+RBC domains used in our analysis of the optimal BTV of Cellpose’s segmentation outputs. Four versions of each domain are pictured: the original SEM image, the manually segmented ground truth, Cellpose’s gradient flow output, and Cellpose’s cell probability output. (PDF) [file pcbi.1012853.s003.pdf]

S3 Text - SUPPORTING FIGURES

The fifteen domains (five sparse, five dense, five dense+RBC) used in our analysis of the optimal BTV of Cellpose's segmentation outputs are shown in Figs. C-E. For each domain type, the figures show, from left to right, the raw SEM image, the manually segmented domain ('ground truth'), the gradient flow map, and the cell probability map. The latter two are Cellpose output.

Sparse Domains

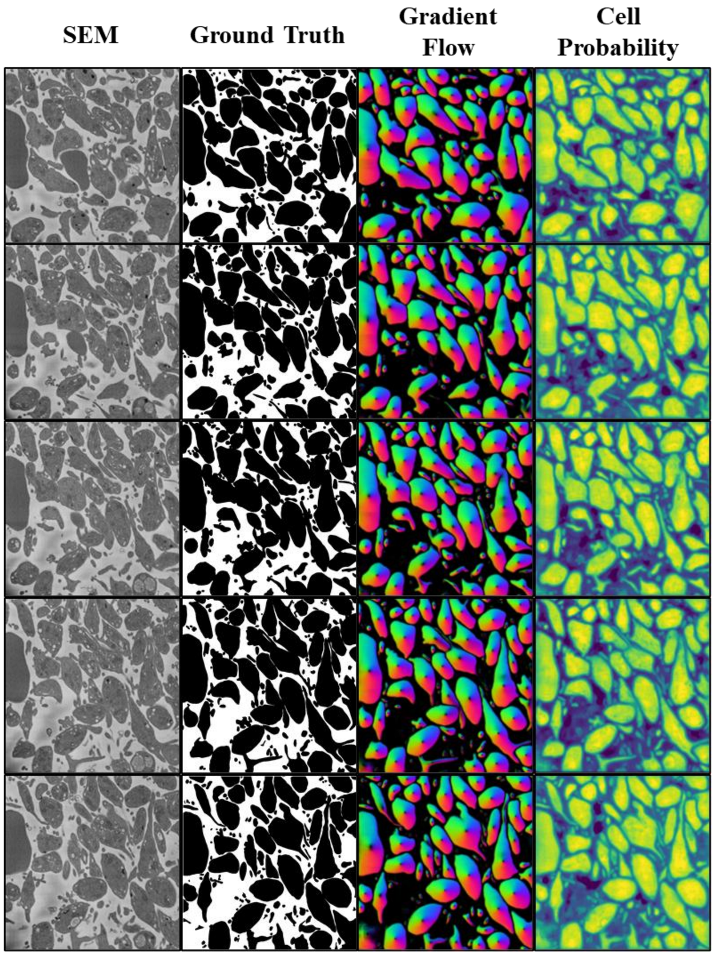

Fig E in S3 Text. The five sparse domains used in our analysis of the optimal BTV of Cellpose's segmentation outputs. Four versions of each domain are pictured: the original SEM image, the manually segmented ground truth, Cellpose's gradient flow output, and Cellpose's cell probability output.

Dense Domains

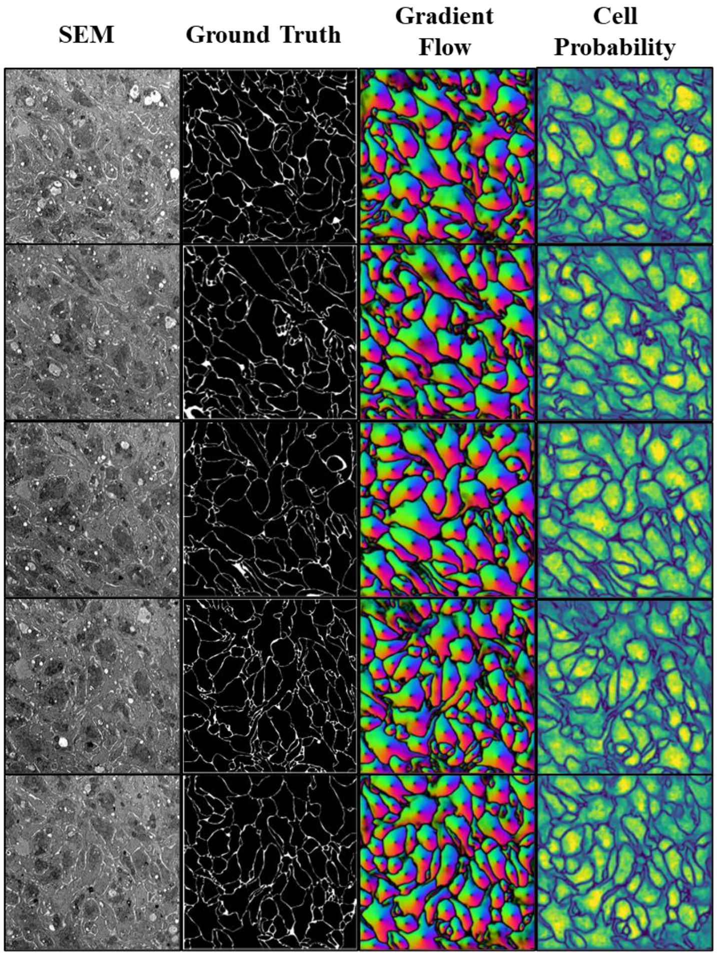

Fig F in S3 Text. *The five dense domains used in our analysis of the optimal BTV of Cellpose's segmentation outputs. Four versions of each domain are pictured: the original SEM image, the manually segmented ground truth, Cellpose's gradient flow output, and Cellpose's cell probability output.*

Dense+RBC Domains

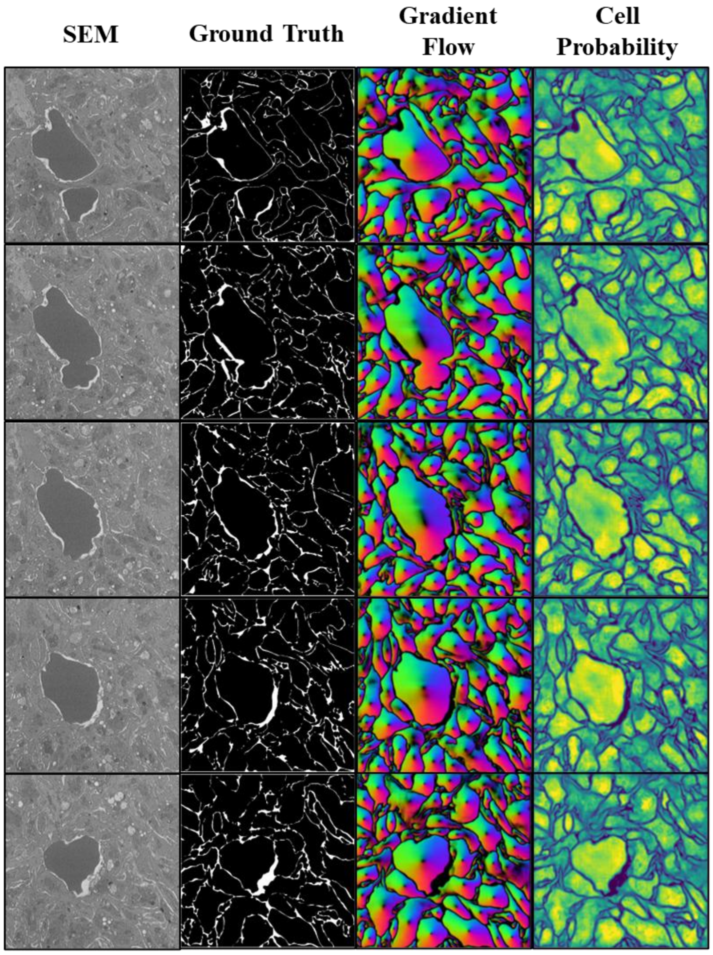

Fig G in S3 Text. The five dense+RBC domains used in our analysis of the optimal BTV of Cellpose's segmentation outputs. Four versions of each domain are pictured: the original SEM image, the manually segmented ground truth, Cellpose's gradient flow output, and Cellpose's cell probability output.
