# Supplementary material for: From imaging to computational domains for physics-driven molecular biology simulations: Hindered diffusion in platelet masses
Source: PLoS Comput Biol. 2025 Jul 7;21(7):e1012853. doi: 10.1371/journal.pcbi.1012853 (PMC12244541; doi:10.1371/journal.pcbi.1012853)
Supplement: Attachment — Submitted filename: Resubmission_letter_PLOS_05.29.2025.docx [file pcbi.1012853.s004.docx]

May 28, 2025

Dear Profs. Zhang and Birtwistle:

I am writing to resubmit our manuscript entitled ‘*From imaging to computational domains for physics-driven molecular biology simulations: Hindered diffusion in platelet masses*’ to address the comments raised by the reviewers. We were pleased to see that the overall reviewer feedback was positive and hope that in responding to the suggestions and comments the manuscript is further improved. We have responded to all the individual comments in the ensuing pages. We look forward to further action on the manuscript.

Sincerely,

Talid Sinno, PhD

**Responses to Reviewer Comments**

**Reviewer #1:**

*This is a well written paper that describes a computational pipeline to quantify diffusion of particles in 2D images that are segmented into cellular matter and surrounding pores. The value of this approach is its technique to quantify estimates of diffusivity in in-vivo imaged data. The authors acknowledge all limitations that came to my mind as I was reading the manuscript. I think this manuscript is worthy of publication after a few minor revisions.*

We thank the referee for their positive comments overall. In the following we address each of the specific suggestions for further improving the manuscript.

*1. The abstract misleads that "large stacks" of 2D images are being used to computationally model a 3D thrombus. in fact, this is why I agreed to review this manuscript. Please better communicate your actual approach.*

Although we do not explicitly state that we are modeling a 3D thrombus in the present paper, upon rereading the abstract, we agree with the referee that our wording might imply this. We have reworded the relevant sentence by removing the term “large stacks”. The new sentence reads:

“We used Serial Block Face – Scanning Electron Microscopy (SBF-SEM) to image select volumes of hemostatic masses formed in a mouse jugular vein, producing high-resolution 2D images.”

We also added the descriptor “2D” in the abstract to make clear what the scope of the present study is.

*2. The motivation and background section is superb.*

We appreciate the positive feedback.

*3. Please clearly indicate in the text (earlier than the limitations section) that your difusion analyses are only for 2D planes.*

Again, upon further consideration, we agree with the referee that this should be explicitly stated. In addition to the modifications in the abstract, we have also included the following in the Introduction section:

“While these image stacks may, in principle, be used to create 3D domains, here we restrict our analysis to 2D by considering a single image at a time.”

*4. 1000 particles diffusing for 0.003 sec seems really coarse and fast. Are the 1000 particle across the entire domain or for each subdomain? Please clarify.*

We agree with the referee that this was somewhat ambiguous. The simulation parameters (1000 particles, 0.0003 s) refer to diffusion calculations for each subdomain. To preserve continuity, we have moved the discussion of the simulation parameters to the Results section “Spatially Resolved Intrathrombus Molecular Diffusivity”. Here, we now also discuss the diffusion results in more detail and refer the reader to a new figure in the SI (Fig. B), which shows example MSD curves justifying our choice of particle number and simulation time. In summary, the time cutoff and particle diffusion coefficient we use for these calculations is sufficiently long for each particle to explore over half of the sub-domain and therefore provide a meaningful estimate of the local diffusivity.

*5.* *Please give an estimate (on average) of how many pixels wide are the typical pore widths in each of the three image types.*

We have now included a short discussion of the pore widths in different regions in the Results section entitled “SBF-SEM Image Segmentation: Visual Accuracy”. The additional text, which also refers to a new Fig. E in the SI that shows the pore width distributions, reads:

“This difference in improvement across different types of domains is not unexpected given the difference in pore geometry. On average, dense regions are characterized by a relatively narrow pore size distribution with a mean pore width of about 40 nm (5 pixels), while sparse regions exhibit pore widths ranging from 40 nm (5 pixels) to 100s of nm (10s of pixels). Quantitative representation of the pore width distributions for different regions are shown in S2 Text, Fig. E. Notably, the long tail of the sparse domain distribution shows that many of the pore widths in sparse domains are quite large and therefore easily resolved with the 8 nm resolution of the SEM imaging.”

*6. Please provide typical CPU times and resources required to perform the CNN training and well as run the heat maps.*

We have purposely left out discussion of computational cost because different runs were performed on different platforms. We plan to provide a systematic analysis of computational cost in future work when we focus on 3D simulations. That said, to respond to the referee’s question, each subdomain diffusion run was executed on a single CPU core (3.0 GHz Intel Xeon) and took about 5-6 minutes to perform. Heatmaps were computed using multiple cores in an embarrassingly parallel mode. Cellpose training per image typically required 0.5 s of runtime on an Nvidia GTX 3060 GPU, i.e., for ~100 SEM images the total training time was about 1 minute.

*7. In Fig. 9, can you add and/or highlight which data points are associated with the ground truth segmented images? The reason is: the reader may question how necessary is it to include the additional CNN segmented images if the 5-15 manually segmented images essentially provide the same linear fit?*

All of the data shown in Fig. 9 is based on Cellpose segmented images and no ground truth labelled images were used. We have now included 30 additional data points (orange squares) computed on randomly selected subdomains from ground truth images in Fig. 9, which show that there is no systematic difference between the ground truth and Cellpose generated images.

*8. The Discussion section is weak. The first 3 paragraphs read like an extended abstract, rehashing things already stated in more detail in the Results section. The latter paragraphs have good discussion content. Please decide how to resolve this. A short Discussion section is fine.*

We have significantly revised the Discussion section to address these concerns. The first paragraph now better transmits the overall mission of the work and some of the specific questions that can be addressed with the modeling framework presented here. The following 3 paragraphs still describe the overall results but now are presented with less quantitative detail, which was already addressed in the RESULTS section, and we now also provide more emphasis on broad insights.

The specific changes to the Discussion section are highlighted in the manuscript version with tracked changes.

*9. Please beef up the "so what?" aspect in your discussion. How does this work generalize to something of future relevance for thrombosis modeling? Perhaps discuss future work and/or forecasting how this work can be synergistically coupled to other modeling approaches.*

We believe that this issue has been addressed by the rewrite discussed in the previous Item 8 above.

**Reviewer #2:**

*I believe that this is the original work of the authors. The quality of the manuscript is good and fulfills the requirements of the journal. This is an interesting observation and there is very little in the literature on this topic. The contents are presented in a clear and narrative manner, which is easy to understand for readers with various background.*

We thank Dr. Razaghi for the positive remarks.

*1. Not much has been said about the importance of this study, and this is the weakness of the article.*

Please see the response to Reviewer #1, Item 8. Specifically, we have rewritten the Discussion section to better address the importance of this toolkit and how it can be used to address questions related to the modeling and understanding of hemostasis and thrombosis.

*2. There is a lack of a general discussion to summarize the results. There is no proper discussion based on the results obtained in the article.*

Again, we believe that the revisions of the Discussion section address this concern. Please see the response to Reviewer #1, Item 8. We would also like to emphasize that the key results of the present work are methodological, i.e., the development and validation of the computational pipeline, which are discussed rather extensively throughout the manuscript. In the revised Discussion section, we have tried to better emphasize that this work serves as the foundation for future studies that will be more focused on specific biological questions.

**Reviewer #3:**

*The manuscript describes a pipelined process to reconstruct from SBF-SEM images the initial conditions (computational domain) for numerical simulations. A lattice kinetic MC method is used to simulate molecule diffusion on these reconstructed computational domains. Although the result of hindered diffusion of molecules through cell packed domains is previously known, using deep learning to help image analysis is quite interesting.*

We thank the referee for the positive remarks.

*1. The raw images, the lattice kinetic MC code are not available.*

We have created a GitHub site where we have deposited the raw images used in this study and the LKMC code used to generate the diffusion data. The link to the GitHub site has been entered during the resubmission process.
